# CORRIGENDUM

**DOI:** 10.1002/mbo3.1305

**Published:** 2022-06-27

Correction: “Absolute quantitation of microbes using 16S rRNA gene metabarcoding: A rapid normalization of relative abundances by quantitative PCR targeting a 16S rRNA gene spike‐in standard” by Olivier Zemb | Caroline S. Achard | Jerome Hamelin | Marie‐Léa De Almeida | Béatrice Gabinaud | Laurent Cauquil | Lisanne M.G. Verschuren | Jean‐Jacques Godon published online on 11 January 2020.

Figure 1 of the original version of published article has been replaced. The correct figure is placed below:

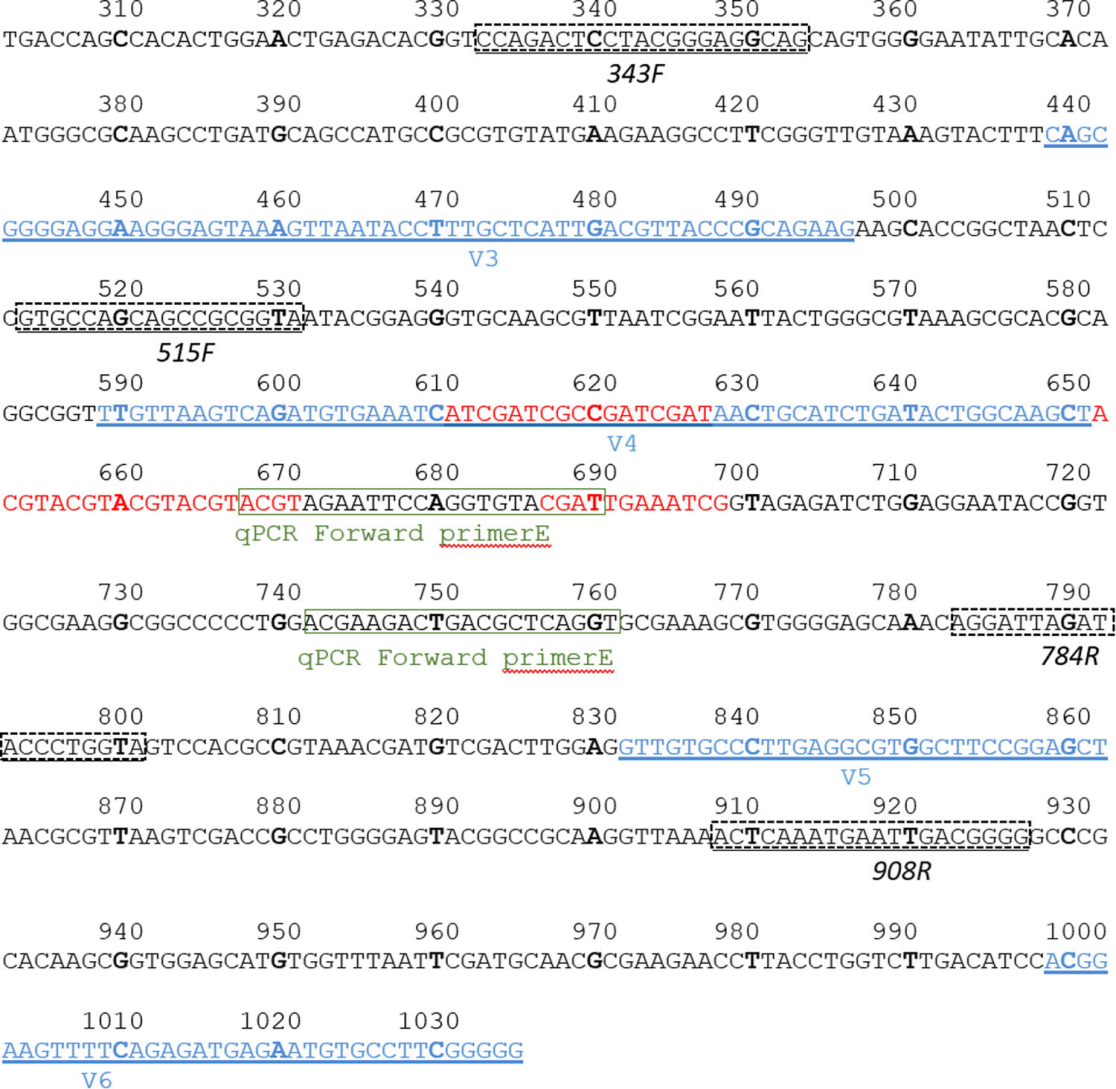

In the original version, in the text (section 2.3) “forward and reverse primers E 5′‐CAGATGTGAAATCATCGATCG/5′‐CCGATTTCAATCGTACACCTG” should read “forward and reverse primers E 5′‐ACGTAGAATTCCAGGTGTACGAT/5′‐ACCTGAGCGTCAGTCTTCGT”.
